# Comparing the response of triple therapy and conventional treatment in male congenital hypogonadotropic hypogonadism: a randomized controlled trial

**DOI:** 10.3389/fendo.2026.1777584

**Published:** 2026-04-01

**Authors:** Biona Devi Konsam, Sanjay Kumar Bhadada, Pinaki Dutta, Ujjwal Gorsi, Mintu Mani Baruah, Trupti Nagendra Prasad, Rama Walia

**Affiliations:** 1 Department of Endocrinology, Postgraduate Institute of Medical Education and Research (PGIMER), Chandigarh, India; 2 Department of Radiodiagnosis, Postgraduate Institute of Medical Education and Research (PGIMER), Chandigarh, India

**Keywords:** AMH, hypogonadotropic hypogonadism, inhibin B, spermatogenesis, testosterone

## Abstract

**Background:**

This study investigated whether triple therapy with human chorionic gonadotropin (hCG), follicle-stimulating hormone(FSH) and testosterone(T) in congenital hypogonadotropic hypogonadism(CHH) promoted more timely virilization, aiding psychosocial development while reducing hCG requirements, offering a balanced approach to long-term management.

**Methods:**

An open-label randomized controlled trial (1:1:1) was conducted in adult males with CHH. Group A received triple therapy, Group B received combined hCG and FSH from the outset, and Group C received hCG monotherapy followed by combined FSH and hCG. Initial doses comprised hCG 2,000 IU twice weekly, FSH 75 IU thrice weekly and intramuscular testosterone(T) 100 mg every two weeks. Group A titrated hCG to achieve AMH of 7.4ng/ml; Groups B and C aimed for T normalization. Primary outcomes were hCG/FSH doses required for spermatogenesis induction and the time to spermatogenesis.

**Results:**

Forty-five CHH males (mean age 25.8 ± 6.1years) were randomized. Spermatogenesis was achieved in 84.6% of group A participants compared with 69.2% and 75% in groups B and C, respectively(p=0.648). Median hCG dose at spermatogenesis was 7500IU/week in group A and 9000IU/week in groups B and C(p=0.016). The time to spermatogenesis was comparable (Groups A/B:12 months; Group C:15 months;p=0.345). Group A participants achieved an AMH of 3.5(2.31-5.38)ng/ml, comparable to the other groups(p=0.962). Predictors of spermatogenesis included USGmTV cut-off of 1.97ml (sensitivity-86.2%,specificity-62.5%), hCG dose of 9,000 IU/week (sensitivity-79.3%,specificity-87.5%) and an Inh B cut-off of 66.8 pg/ml(sensitivity-92.6%,specificity-100%).

**Conclusions:**

Triple therapy provided a better quality of life without compromising spermatogenesis. The AMH and Inh B provided an effective means of monitoring.

**Clinical trial registration:**

www.ctri.nic.in, identifier CTRI/2022/05/042795.

## Introduction

Male hypogonadism is a condition characterized by insufficient testosterone production and subfertility. Hypogonadotropic hypogonadism (HH) is a form of hypogonadism in which gonadal insufficiency occurs due to a deficiency of pituitary gonadotropin levels. HH can arise from dysfunction at the hypothalamic level, where there is absent or insufficient secretion of gonadotropin-releasing hormone (GnRH), or at the pituitary level, where there is a primary failure in gonadotropin secretion (1). HH can be either acquired or congenital. Congenital HH (CHH) comprises patients with (Kallmann syndrome) and without anosmia, but patients with congenital hypopituitarism encounter similar issues (2). Acquired causes of HH can either be pathological, including parasellarinfections, infiltrative diseases, iron overload, tumors, irradiation, surgery and traumatic brain injury, or functional, due to extremes of body mass, opiates, hyperprolactinemia, or chronic systemic diseases. CHH constitutes around 1% of male infertility. The CHH and congenital hypopituitarism affect around 1 in 4,000 and 1 in 10,000 respectively. Regardless of the cause, HH is one of the causes of male subfertility that is amenable to treatment with hormone replacement (3).

The classical fertility treatments of male HH includes human chorionic gonadotropin (hCG), which acts on the same LHCG receptor as LH, but has a much longer half-life, and follicle-stimulating hormone (FSH), whether urinary (e.g. hMG for FSH) or recombinant. Pulsatile GnRH is only effective where the defect is hypothalamic and is available in only a few countries. There are various treatment regimens, one of which begins with a phase of hCG monotherapy followed by the addition of FSH or (4)., or by combining hCG and FSH/HMG from the outset, and this has been shown to give better results (5). The beneficial effect was evidenced by better sperm counts, higher inhibin B (Inh B) levels, and reduction in anti-mullerian hormone (AMH) (6).

The hCG leads to a rise in intratesticular testosterone (ITT), reaching approximately 100 times more than systemic T. Further, spermatogenesis is an early event in puberty, which again corresponds to the above fact. However, the dose of hCG necessary for initiation of spermatogenesis is less than that required for the achievement of normal circulating T concentrations and virilization. Indeed, it has been hypothesized that the dose needed for virilization might be associated with damage to the seminiferous tubule compartment (hyalinization) (7–9). Moreover, it can take weeks or even months of adjustments to the hCG dose before adequate T levels are achieved, during which time treatment-naive males may take longer to achieve complete virilization and those who had previously been taking testosterone might experience sexual dysfunction and fatigue. Adding exogenous T to the combination therapy of hCG and FSH in complete HH does not raise concerns about suppressing the hypothalamic-pituitary-testicular (HPT) axis, given that the participants already exhibit complete axis disruption (10). Hence, utilizing lower doses of hCG along with parenteral T preparations and FSH might equally achieve spermatogenesis whilst completing and faster virilization and with lower risk of hyalinization. Although traditional monitoring of hCG dose with T and E levels is not possible when concomitant exogenous testosterone is given, studies have shown that ITT correlates with spermatogenesis, and the rise in ITT leads to fall in AMH as maturing Sertoli cells switch to secreting Inhibin B instead. Therefore, AMH, an inverse surrogate marker of ITT, was explored as a monitoring modality (11).

This study was proposed to compare the response of combined hCG, FSH, and T(Triple therapy) to conventional hCG combined with FSH in treating patients with CHH. The emergence of Inh B and AMH as monitoring modalities was also addressed in this study.

## Methodology

This study was an open-label randomized controlled trial conducted in a tertiary care center in Northern India from May 2022 to March 2024. The study was conducted in alignment with the Declaration of Helsinki. The Institutional Ethics Committee approved the study and registered it in the Clinical Trials Registry-India (CTRI/2022/05/042795).

### Participants

All male patients who presented with delayed puberty were screened. The diagnosis of CHH was based on symptoms of hypogonadism with testicular volume (TV) of less than 4ml and laboratory parameters of low plasma luteinizing hormone (LH), FSH and T. The GnRH stimulation test, FSH and GnRH-stimulated Inh B were performed to assist in establishing the diagnosis (12, 13). Comprehensive genetic testing could not be conducted in all participants because of financial constraints. Unwilling participants, acquired HH, functional HH, primary hypogonadism, cryptorchidism, congenital hypopituitarism on recombinant growth hormone (rGH) therapy, prior treatment with gonadotropin and participants with raised aminotransferases were excluded from the study.

The sample size was estimated based on previously reported spermatogenesis rates of approximately 65–85% between comparable treatment groups. Assuming a non-inferiority margin of 20%, with 80% power and a two-sided 95% confidence interval (CI), the minimum required sample size was calculated to be 14 participants per group. To mitigate potential attrition, we opted to include 15 subjects per group, yielding a total of 45 participants (14).

### Study procedure

**Figure 1:**
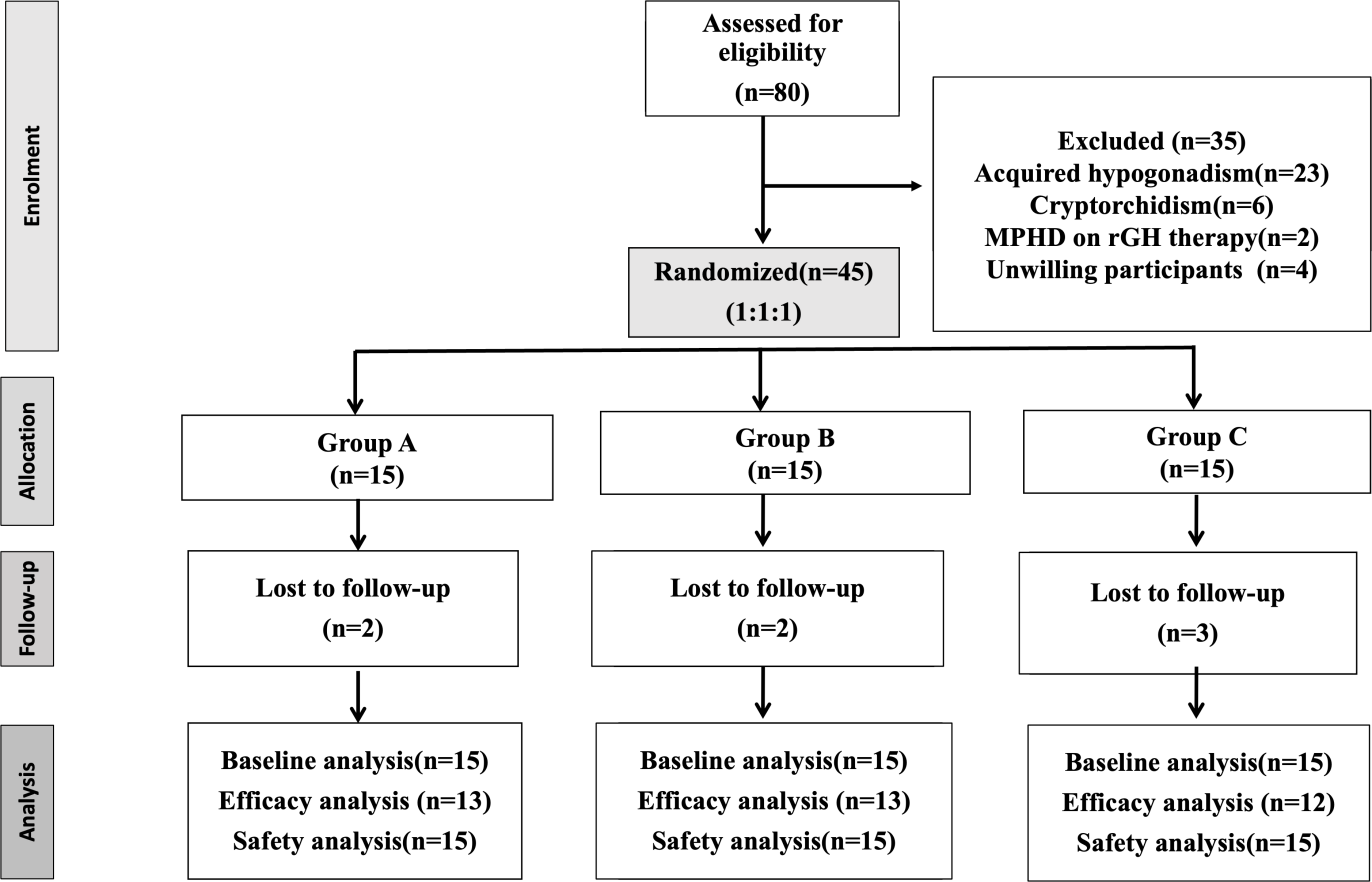
Trial profile.

The participants were randomized (1:1:1) into three groups using a random allocation sequence in a permuted block of three (**Figure 1**). Group A participants received the triple therapy (hCG, FSH and testosterone), group B received a combination of hCG and FSH, and Group C received hCG alone till normalization of T followed by the addition of FSH. The starting doses of hCG, FSH and T were 2,000 IU twice weekly, 75 IU thrice weekly and 100mg every 2 weeks respectively. The hCG dosage was titrated to reduce AMH to 7.4 ng/ml in group A, and normalization of plasma T(9.0-27.8nmol/L) in groups B and C, respectively. The AMH target was based on the interim result from a previous study, which identified a value of 7.4 ng/ml as a predictor of spermatogenesis (14). In all the groups, FSH was targeted at 4–8 mIU/ml (15). In group A, T dose titration was done to achieve a nadir plasma T in the normal range. The maximum doses of hCG and FSH were 5,000IU thrice weekly and 225 IU thrice weekly, respectively. Successful spermatogenesis was defined as the presence of a sperm concentration of at least 1 × 10^6^/mL on semen analysis, with repeat sampling done after 2 weeks to account for variability.

Baseline virilization by body hair score (BHS), upper/lower segment (U/L) ratio, height(cm), weight(Kg) and body mass index(BMI) were noted for all the subjects. Sexual functioning was evaluated using the Sexual Desire Inventory-2(SDI-2), while the quality of life (QoL) was assessed through the Pubertal Development Scale(PDS) and the Quantitative Androgen Deficiency in Aging Men(qADAM) questionnaire (**Supplementary Appendix**). The muscle strength was assessed by measuring handgrip strength (HGS) with the help of the Jamar Plus Digital Hand Dynamometer (Jamar^®^, Patterson Medical).

The BHS is a scoring system obtained from the modification of Ferriman and Gallwey (1961) and Tanner (1962) criteria. The scoring of the body hair was done in 13 body regions in a semiquantitative method. For each region, the scoring of hair densities ranges from 0 (absence of hair) to 4 points (maximum hair growth). The total score was defined as the sum of the body region scores (16).

The SDI-2 is a self-administered questionnaire for assessing sexual desire. It is a self-report measure including 14 items that give information about a person’s interest in an opposite partner (dyadic) and self (solitary) sexual activity. By combining both, an overall measure of sexual desire was calculated. The total score ranged from 0 to 109. Items 1–9 measure dyadic desire, and items 10–14 measure solitary desire (17).

The PDS was used to assess the development of secondary sexual characteristics (18). Physical changes associated with puberty have an impact on the social and psychological development of an adolescent. Hence, quality of life was assessed using this scale.

The qADAM questionnaire is a tool for quantifying the severity of hypogonadism. The qADAM questionnaire consisted of 10 questions of the Saint Louis University ADAM questionnaire, where a Likert scale of 1–5 was used for each question, in which 5 denoted no symptom and 1 represented the maximal symptoms. All questions are given equal weightage. The sum of these gave a total qADAM score ranging from 10 to 50. A score of 10 was considered to be the most symptomatic and 50 is the least symptomatic (19).

Endocrine investigations included plasma LH, FSH, T, AMH, estradiol (E2), cortisol, thyroid function test, and prolactin, which were measured using electrochemiluminescence immunoassay, ECLIA (Elecsys 2010 Analyzer, Roche Diagnostics, Mannheim, Germany). The dynamic range of AMH is 0.01–46 ng/ml with a lower limit of detection (LOD) of 0.01ng/ml. The inter-assay CV was 3.5% and the intra-assay CV was 1.7%. Serum Inh B was measured by an enzyme-linked immunosorbent assay(ELISA) kit (Catalog # AL-107, RRID: AB_2783661). The dynamic range is 3.9–542 pg/ml with a lower LOD of 1.6 pg/ml. The inter-assay CV was 3.05-6.32% and the intra-assay CV was 4.99%. Testicular volumes (TV) by the Prader Orchidometer and Ultrasound and Bone Mineral Density by dual-energy X-ray absorptiometry (DXA) scan were performed using HOLOGIC Discovery A (QDR 4500; Hologic, Inc., Bedford, MA). The mean TV (mTV) were calculated and used for further reference and assessment of TV.

The study was conducted over a period of 18 months excluding 4 months of enrolment period.

The T, AMH, FSH, E2 and Inh B were monitored every 6 weeks and USG TV and semen analysis were done every 3 months. The drug dose titration was done every 6 weeks and clinical parameters were assessed quarterly. The handgrip strength and DXA scan were repeated after 12 months.

The primary objectives were to compare the doses of hCG and FSH required for the induction of spermatogenesis and the time to achieve spermatogenesis. The secondary objectives were to assess the improvement in virilization by using BHS, sexual functioning by SDI-2 and QoL by PDS and qADAM questionnaire after one year. The various predictors of spermatogenesis were analyzed which included baseline clinical and biochemical parameters and parameters at the time of spermatogenesis.

### Statistical analysis

The intention-to-treat analysis was employed to calculate primary outcomes, while per-protocol analysis was utilized for safety outcomes. After checking for normality of the distribution, data were presented as absolute numbers, mean, median, standard deviation (SD) and interquartile ranges, or percentages. Categorical variables were presented either as a number or as a percentage (%) while continuous variables were denoted as a mean ± SD. Inter-group and Intra-group analyses of baseline and follow-up parameters were conducted using Chi-Square or Fisher’s Exact test for categorical data, and Mann-Whitney U or Kruskal Wallis H and Wilcoxon Signed rank/Friedman ANOVA test for continuous variables for non-parametric data.

A binary logistic regression analysis was carried out to find predictors of successful spermatogenesis. Receiver Operating Characteristic (ROC) curves were generated to determine the cut-off values for relevant parameters. All analyses were performed using the statistical package for the Social Sciences (SPSS) 26 version. A significance level of p<0.05 was considered significant.

## Results

Initially, a total of 80 participants were screened for the inclusion criteria. After excluding 35 participants (acquired hypogonadism=23, cryptorchidism=6, congenital hypopituitarism on rGH therapy=2 and unwilling participants=4), 45 underwent randomization into 3 groups with 15 in each group. Two participants from groups A and B and three from group C were lost to follow-up (**Figure 1**).

The mean age of the participants was 25.8 ± 6.14 years. The mean age(p=0.996) and BMI(p=0.104) were comparable among all the groups. Anosmia/hyposmia and synkinesia were observed in 15.6% and 11.1% respectively. The median of the clinical parameters like BHS(p=0.769), PDS(p=0.157), qADAM(p=0.205) and SDI-2(p=0.524) were comparable in all the groups. The baseline mTV of the three groups was 2(1-3) ml (p=0.821). Kallmann syndrome and congenital hypopituitarism were observed in 24.4% and 20% respectively with MRI abnormality noted in 40% of the total participants. The prior testosterone therapy was received by 35.6% of the participants and all underwent a washout period of 6 weeks before enrolling into the study. The FSH at baseline for groups A, B and C were 0.83(0.33-1.02) mIU/ml, 0.65(0.37-1.28) mIU/ml and 0.54(0.3-0.69) mIU/ml respectively (p=0.132). The other baseline hormonal parameters were comparable among the groups. Median baseline USG mTV in groups A, B and C were 0.63(0.0.33-1.3)ml, 0.53(0.2-1.13)ml and 0.67(0.53-0.73)ml respectively, which were comparable(p=0.88) (**Table 1**). The time profile graphs for T, FSH, AMH, and InhB are presented in **Supplementary Figure 1**.

**Table 1:** Baseline demographics, clinical and laboratory parameters.

Parameters	Total (n=45)	Group A (n=15)	Group B (n=15)	Group C (N = 15)	P-value
Age (years)	25.8 ± 6.14	26.1 ± 7.26	25.4 ± 6.39	25.9 ± 6.20	0.996
Height (cm)	169.9 ± 8.42	169 ± 8.42	169.0 ± 7.43	172.2 ± 9.78	0.513
Weight (kg)	64.7 ± 14.9	65.6 ± 13.5	58.3 ± 12.8	70.5 ± 16.5	0.077
BMI (kg/m^2^)	22.4 ± 4.72	22.9 ± 3.81	20.3 ± 3.95	23.8 ± 5.56	0.104
BHS	8 (6-12)	8 (5-14)	8 (5-12)	10 (7-12)	0.769
PDS	7 (6-8)	7 (5-8)	7 (5-8)	8 (7-9)	0.157
qADAM	23 (21-24)	23 (21-25)	24 (22-25)	22 (19-24)	0.205
SDI-2	24 (19-33)	23 (19-32)	25 (17-33)	25 (22-36)	0.524
mTV (ml)	2 (1.25-3)	2 (1-3)	2 (1.5-3)	2 (1-3)	0.821
Kallman syndrome (%)	11 (24.4)	4 (26.7)	5 (33.3)	2 (13.3)	0.522
MPHD (%)	9 (20)	4 (26.7)	3 (20.0)	2 (13.3)	0.522
MRI abnormality (%)	16 (40)	7 (53.8)	6 (40)	3 (20)	0.086
Prior T therapy (%)	16 (35.6)	5 (33.3)	4 (26.7)	7 (46.7)	0.569
HGS (kg)	20.5 ± 6.32	18.5 ± 4.17	23.2 ± 7.43	19.6 ± 6.24	0.19
Hemoglobin (g/dl)	12.6 ± 1.17	12.7 ± 0.8	12.9 ± 3.24	13.0 ± 1.09	0.088
AST (U/ml)	23.8 (20.6-32)	27.7 (21.4-49.2)	27 (20.9-32)	23.8 (19.3-29.3)	0.435
ALT (U/ml)	23.7 (17.2-35.2)	28 (18-45.8)	23.7 (18-31.8)	22.1 (14.8-32)	0.365
Creatinine (mg/dl)	0.7 ± 0.14	0.67 ± 0.10	0.68 ± 0.13	0.77 ± 0.15	0.097
FSH (mIU/ml)	0.53 (0.3-1.02)	0.83 (0.33-1.02)	0.65 (0.37-1.28)	0.54 (0.3-0.69)	0.132
LH (mIU/ml)	0.3 (0.3-0.5)	0.3 (0.3-0.42)	0.3 (0.3-0.93)	0.34 (0.3-0.5)	0.728
T (nmol/L)	0.27 (0.09-0.76)	0.32 (0.87-0.77)	0.25 (0.08-0.32)	0.43 (0.09-0.72)	0.467
AMH (ng/ml)	19.2 (12.5-19.2)	18.4 (12-25.1)	20.3 (8.43-23)	18.2 (13.7-25.3)	0.945
Inh B (pg/ml)	16 (10.6-23)	15.5 (12.3-21.4)	16 (10.6-23)	16.5 (10.2-26.7)	0.962
E2 (pg/ml)	5 (5-5)	5 (5-5)	5 (5-5)	5 (5-5)	_
Prolactin (ng/ml)	7.3 (4.12-9.74)	8.26 (4.11-10.5)	7.18 (5.26-9.74)	6.29 (3.36-9.46	0.395
USG mTV (ml)	0.65 (0.35-0.98)	0.63 (0.33-1.3)	0.53 (0.2-1.13)	0.67 (0.53-0.73)	0.88
BMD FN (g/cm^2^)	0.732 ± 0.976	0.694 ± 0.105	0.704± 0.088	0.776± 0.088	0.769
BMD L1-4 (g/cm^2^)	0.765 ± 0.105	0.727 ± 0.124	0.748± 0.090	0.83 ± 0.093	0.106
BMD Rad1/3 (g/cm^2^)	0.610 ± 0.833	0.595 ± 0.088	0.574 ± 0.078	0.664 ± 0.055	0.115

BMI, body mass index; BHS, body hair score; PDS, pubertal developmental scale; qADAM, quantitative androgen deficiency in aging men questionnaire; SDI-2, sexual desire inventory-2; mTV, mean testicular volume; MPHD, multiple pituitary hormone deficiency; MRI, magnetic resonance imaging; T, testosterone; HGS, handgrip strength; AST, aspartate aminotransferase; ALT, alanine aminotransferase; FSH, follicle-stimulating hormone; LH, luteinizing hormone; AMH, anti-Mullerian hormone; Inh B, inhibin B; E2, estradiol; USG mTV, mean ultrasound testicular volume; BMD, bone mineral density (FN femur; L1–4 lumbar 1 to 4; Rad 1/3 radius lower one third). 1ng/ml=1mcg/L.

Spermatogenesis was achieved in 29 out of 38 participants constituting 76.3%, with the highest proportion in group A (n=11) 84.6%, compared with 69.2% in group B (n=9) and 75% in group C (n=9) (p=0.648 = ns). The median semen volume was 1.5(1-3)ml, and the median total sperm concentration achieved was 8 x10^6^ (2.8-17.3)/ml. Normal morphology was observed in 50(30-70)% while motility in 20(10-30)%.

The median hCG dose was 7,500 IU/week in Group A at the time of spermatogenesis which was lower than in the other groups (9,000IU/week, p=0.016). The median time to achieve spermatogenesis was comparable with 12 months in groups A and B and 15 months in group C (p=0.345 = ns) (**Supplementary Table 1**). Four participants were seeking biological fatherhood and two of them achieved pregnancy. As expected, group A participants achieved normal median T by 3 months [20.9 (7.5-25.4)nmol/L]. The median T in Group B [15.1(8.8-26)nmol/L] and C[9.9(4.6-16.6)nmol/L] participants were normalized only after 9 months of treatment.

**Figure 2:**
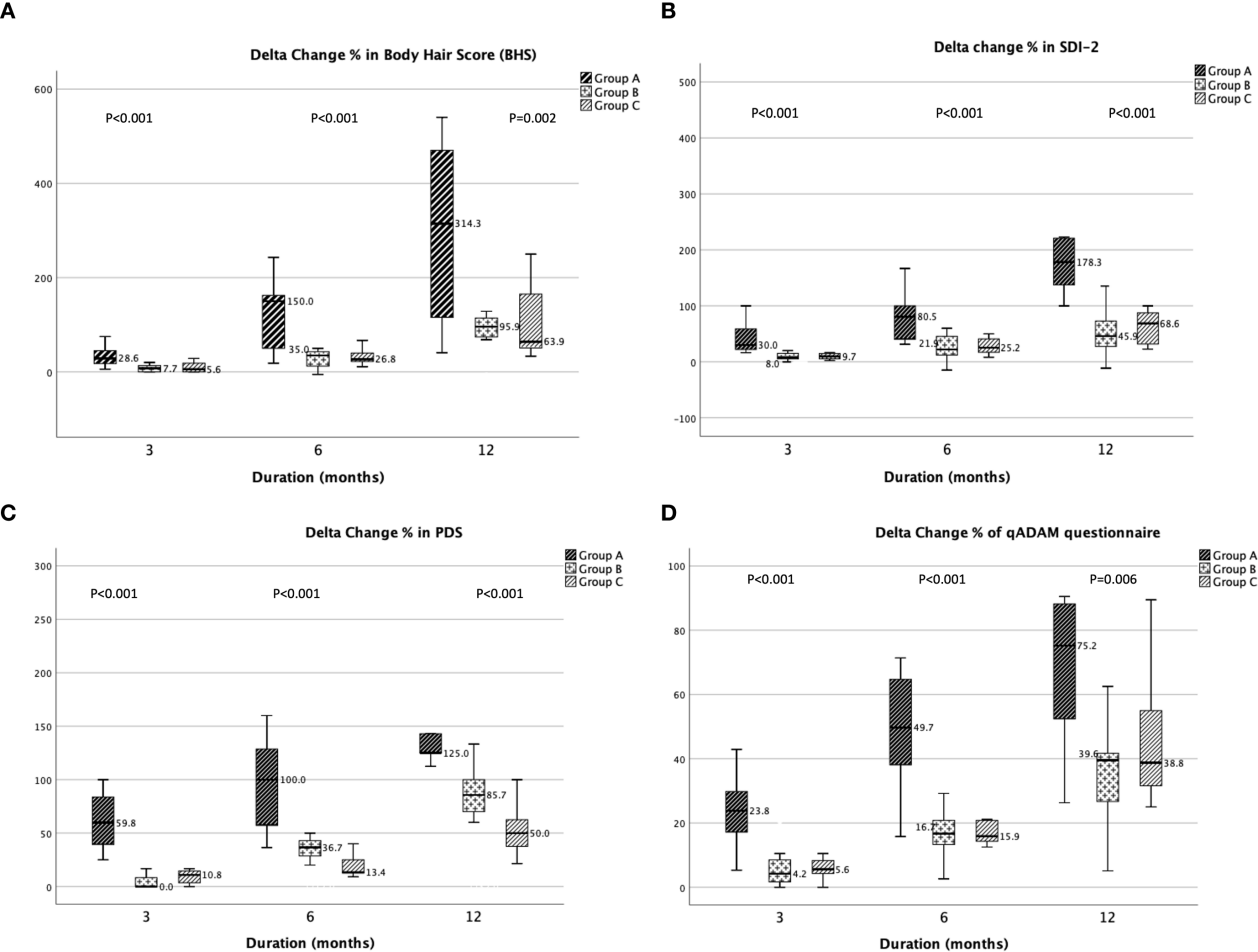
Box plot showing the interval change in **(A)** body hair score (BHS), **(B)** sexual desire inventory-2 (SDI-2), **(C)** pubertal developmental scale (PDS) **(D)** quantitative androgen deficiency in aging men questionnaire (qADAM).

Improvement in virilization was objectively measured with BHS. By 3 months, there was 28.6% increase in BHS in group A patients while in group B and C, the improvements were only 7.7% and 5.6% respectively (p<0.001). By the end of one year, the increase was 314.3% in group A participants, which was significantly more than the other groups (p<0.001) (**Figure 2A**). For Sexual functioning, improvements in SDI-2 were 30%, 8% and 9.7% in group A, B and C patients respectively at 3 months (p<0.001). By one year, there was a 178.3% improvement in group A participants, while the increase in groups B and C was only 45.9% and 68.6% respectively (p<0.001) (**Figure 2B**). The QoL was assessed using the PDS and qADAM questionnaire. For PDS, there was a 59.8% increase at 3 months, which increased to 125% at one year in group A participants (**Figure 2C**). While assessing the qADAM questionnaire, the improvement observed in group A participants was 23.8% in 3 months (p<0.001) and 75.2% (p<0.001) in one year (**Figure 2D**). The absolute improvement in secondary outcomes was shown in **Supplementary Table 2**.

At the time of spermatogenesis, the USG mTV in group C was 2.13(1.97-3.23) ml which was lowest compared to groups A [3.79(1.75-5.32)ml] and B [4.32(3.34-6.17)ml] (p=0.016). The median T in groups A, B and C patients were 23.5(15.8-28.3) nmol/L, 18.8(11.7-27.2) nmol/L and 12.4(9.56-26.5) nmol/L respectively (p=0.261). Plasma AMH levels were 3.5(2.31-5.38) ng/ml, 2.97(2.69-5.54) ng/ml and 3.82(1.95-4.17) ng/ml in groups A, B and C patients respectively (p=0.962). Serum Inh B levels were highest in group A participants but were not significant. (p=0.544) (**Table 2**).

**Table 2:** Comparison of parameters at spermatogenesis among groups.

Group	A (n=11)	B (n=9)	C (n=9)	p value
Age (years)	26.2 ± 7.07	25.2 ± 7.85	26 ± 5.89	0.895
BMI (kg/m^2^)	22.1 ± 3.2	20.8 ± 4.02	23.1 ± 5.69	0.914
FSH (mIU/ml)	3.21 (2.72-3.5)	3.9 (2.11-4.02)	2.9 (1.69-4.49)	0.857
T (nmol/L)	23.5 (15.8-28.3)	18.8 (11.7-27.2)	12.4 (9.56-26.5)	0.261
AMH (ng/ml)	3.5 (2.31-5.38)	2.97 (2.69-5.54)	3.82 (1.95-4.17)	0.962
Inh B (pg/ml)	128.7 (68.5-238)	115 (44.3-278.8	89.2 (63.1-129)	0.544
E2 (pg/ml)	49.3 (41.7-67.5)	37 (16.7-66)	25.8 (14.9-56.4)	0.585
mTV (ml)	8 (6-8)	8 (7.5-11.5)	8 (6-9)	0.189
USG mTV (ml)	3.79 (1.75-5.32)	4.32 (3.34-6.17)	2.13 (1.97-3.23)	**0.016**

Bold values indicate statistically significant results (p<0.05)

Participants with prior testosterone therapy had spermatogenesis in 81.3% when compared to those who were T naïve (55.3%), but the finding was not statistically significant (p=0.155). Age was older in the prior testosterone group, and the result was significant (29.5 ± 5.98 vs 24 ± 5.98, p=0.006). The rest of the parameters failed to yield any significant observations (**Supplementary Table 3**).

While exploring the predictors of spermatogenesis, we did not observe any significant difference in age (p=0.761) and BMI (p=0.973). No significant difference was observed with baseline parameters including plasma LH (p = 0.519), FSH (p = 0.248), T (p = 0.107), AMH (p = 0.794), Inh B (p = 0.741) and E2 (p=0.931). There was also no significant difference in mTV (p = 0.379) and USG mTV (p = 0.277) at baseline. Except for Inh B, other hormonal parameters at the end of the study did not show any statistically significant differences. The Inh B level at spermatogenesis was 129(89.2-238) pg/ml while those who did not achieve spermatogenesis had a level of 25.9(9.58-60.3)pg/ml (p<0.001). However, the USG mTV was significantly higher in the spermatogenesis group compared to the group that did not achieve spermatogenesis (p=0.026). The hCG dose was significantly lower in the spermatogenesis group (9,000 vs 15,000 IU/week) (p = 0.004) (**Table 3**).

**Table 3:** Comparison of participants in relation to spermatogenesis.

Spermatogenesis		Yes (n=29)	No (n=9)	p value
Baseline parameters	Age (years)	25.8 ± 6.75	25 ± 5.87	0.761
	BMI (kg/m^2^)	22 ± 4.29	22.4 ± 6.08	0.973
	LH (mIU/ml)	0.3 (0.3-0.5)	0.3 (0.3-0.49)	0.519
	FSH (mIU/ml)	0.7 (0.32-1.13)	0.37 (0.3-0.62)	0.248
	T (nmol/L)	0.32 (0.11-0.74)	0.09 (0.08-0.37)	0.107
	AMH (ng/ml)	18.4 (11.5-25)	18.9 (9.42-30.7)	0.794
	Inh B (pg/ml)	15.8 (11-22.8)	18 (7.03-29.3)	0.741
	E2 (pg/ml)	5 (5-5)	5 (5-5)	0.931
	mTV (ml)	2 (1.5-3)	2 (1-2.75)	0.379
	USG mTV (ml)	0.67 (0.4-0.98)	0.52 (0.24-1.01)	0.277
	hCG dose (IU/week)	4000 (4000-4000)	4000 (4000-4000)	1
	FSH dose (IU/week)	225 (225-225)	225 (225-225)	1
Follow-up parameters	FSH (mIU/ml)	3.2 (2.3-3.96)	2.55 (1.45-3.89)	0.336
	T (nmol/L)	18.8 (12-26.4)	18.9 (9.69-22.5)	0.519
	AMH (ng/ml)	3.57 (2.6-4.98)	2.87 (1.89-8.6)	0.802
	Inh B (pg/ml)	129 (89.2-238)	25.9 (9.58-60.3)	**<0.001**
	E2 (pg/ml)	45 (19.4-57.6)	26 (4.9-41.5)	0.068
	mTV (ml)	8 (6.5-9)	5.5 (4.25-8.75)	0.094
	USG mTV (ml)	3.23 (2.07-4.65)	1.87 (1.41-3.35)	**0.026**
	hCG dose (IU/week)	9000 (7500-9000)	15000 (10500-15000)	**0.004**
	FSH dose (IU/week)	450 (450-450)	450 (450-450)	0.893

Bold values indicate statistically significant results (p<0.05).

Univariate Logistic Regression analysis on USG mTV, hCG dose and Inh B at follow-up predicted the likelihood of successful spermatogenesis. At the same time, other parameters, including T, AMH, mTV, and duration of therapy, were also unable to predict. Hence, ROC curves were derived for USG mTV, hCG dose and Inh B. The USG mTV cut-off of 1.97 ml had a sensitivity of 86.2% and a specificity of 62.5% [area under the curve (AUC)=0.759; 95% CI 0.57-0.95;p=0.027] (**Supplementary Figure 2A**). Similarly, the hCG dose of 9000 IU/week had a sensitivity of 79.3% and a specificity of 87.5% [AUC = 0.823;95% CI 0.66-0.99; p=0.006] (**Supplementary Figure 3B**). The Inh B cut off of 66.8 pg/ml had a sensitivity of 92.6% and a specificity of 100% [AUC 0.968; 95% CI 0.90–1.00; p < 0.001]. (**Supplementary Figure 4C**).

### Side effects

The most common side effect was gynecomastia (20%) and the maximum was observed in group B participants (26.7%) and least in group A (13.3%). One participant in group A complained of acne and another participant had an allergic reaction to drugs. Median hematocrit of 41.1(38.6-42) % was observed at the time of spermatogenesis, not requiring any treatment discontinuation. All the groups have similar hematocrit [group A 42(39.6-42), group B 41.1(38-42.2), group C 43.5(40.5-46.4), p=0.06].

## Discussion

This study evaluated the effectiveness of different therapeutic regimens in inducing spermatogenesis among individuals with CHH. Out of 38 participants, 76.3% achieved spermatogenesis, with the highest rate observed in group A (triple therapy) at 84.6%, although not statistically significant (p=0.648). The initial mTV was 2 ml across all groups, indicating congenital onset (p=0.821). Group A required a lower median dose of hCG (7500 IU/week) compared to groups B and C (9000 IU/week) (p=0.016), suggesting enhanced response with triple therapy. The median time to spermatogenesis was shorter in groups A and B (12 months) compared to group C (15 months), though this difference was not significant (p=0.345). Significant clinical improvements were noted in group A. By the end of one year, group A exhibited a 314.3% increase in virilization (BHS), a 178.3% improvement in sexual functioning (SDI-2), and substantial gains in quality of life (125% by PDS and 75.2% by qADAM). The HGS also improved significantly in group A (p=0.003). Key predictors of spermatogenesis included a USG mTV cut-off of 1.97 ml (sensitivity 86.2%, specificity 62.5%), an hCG dose of 9000 IU/week (sensitivity 79.3%, specificity 87.5%) and Inh B cut-off of 66.8 pg/ml(sensitivity 92.6% and specificity 100%). The study indicated that triple therapy is more effective than conventional treatment in achieving virilization and the biological outcomes associated with this, while both approaches are equally effective in achieving spermatogenesis. The findings supported the use of triple therapy as a preferred treatment approach, although further research with larger sample sizes and longer follow-ups is needed to confirm these results.

The management of CHH has explored various treatment options to induce puberty and spermatogenesis, including GnRH, T, hCG, hMG, highly purified urinary derivatives, recombinant FSH, and recombinant LH (20). Traditionally, the induction of spermatogenesis has involved the administration of hCG followed by FSH, although recent approaches advocate for the simultaneous administration of hCG and FSH (21). Reported spermatogenesis rates across different treatments range from 77% to 84%, which included studies where partial HH were included (3, 21, 22). However, conventional treatments have raised concerns regarding the delayed onset of pubertal changes and virilization, which can adversely affect psychological well-being. While lower doses of hCG are sufficient for inducing spermatogenesis, higher doses are necessary for achieving adequate virilization (3, 5, 11, 21, 22). Prolonged high-dose hCG treatment, however, has been linked to potential damage to the seminiferous tubules.

Managing CHH is challenging due to the lengthy and costly treatment, often leading to patient dropout. In response to these challenges, this study introduced a novel triple therapy approach combining hCG, FSH, and T. The response to triple therapy was compared with conventional treatments, whether sequential or combined. Triple therapy demonstrated an impressive spermatogenesis rate of 84.6%, surpassing outcomes from earlier studies and showing no compromise in effectiveness. While no statistically significant difference was demonstrated (p = 0.648), the observed numerical variation in spermatogenesis rates (84.6% vs. 69.2% vs. 75%) may indicate a clinically relevant trend. However, the triple therapy resulted in the highest spermatogenesis rate without any significant difference in the time taken to achieve it when compared to conventional treatments. The protocol combines hCG and FSH to effectively induce spermatogenesis and masculinization in HH patients. The hCG is more potent than LH, as demonstrated by *in vitro* studies showing higher cAMP production, steroidogenesis, and a longer half-life (30 hours vs. 30 minutes for LH), leading to prolonged effects (23). However, high doses of hCG may have counterproductive effects. Excessive hCG can cause LH receptor down-regulation and elevated intratesticular progesterone levels, which can impair spermatogenesis. Thus, optimizing hCG dosage is essential to maximize its benefits while minimizing adverse effects, such as receptor down-regulation and increased intratesticular estradiol (24). By carefully balancing the dosages of hCG, FSH, and possibly testosterone, spermatogenesis can be stimulated effectively while mitigating risks associated with excessive gonadotropin exposure. Additionally, the higher spermatogenesis rate observed may be attributed to the exclusion of participants with cryptorchidism, a known negative predictor of treatment success. These findings suggest that triple therapy offers a promising and potentially more effective approach to managing CHH. Furthermore, normalization of serum testosterone is faster in group A as compared to groups B and C, thus resulting in better virilization, as shown in the time profile figure below (**Supplementary Figure 1A**). It addressed both the need for sufficient spermatogenesis and the timely onset of pubertal changes and virilization, thereby potentially improving the overall treatment experience and outcomes for patients with CHH.

The primary outcome was to compare the hCG dose in all the groups at the time of spermatogenesis. Notably, the group A required the lower hCG dosage (7500 IU/week compared to 9000 IU/week), a significant finding. The reason for the reduced hCG dosage needed to stimulate spermatogenesis lies in the fact that only a minimal amount of hCG is necessary to maintain intratesticular testosterone (ITT) (11). The actions of hCG within the testes occur via the androgen receptor (AR) present on peritubular, Leydig, and Sertoli cells. ITT levels are approximately 100 times higher than serum testosterone levels in normal men, crucial for maintaining spermatogenesis and preventing meiotic arrest at the round spermatid stage. It also inhibits germ cell apoptosis. Spermatogenesis remains unaffected even if ITT was reduced by 50-60% (11). Therefore, even with a lower hCG dose, the process of spermatogenesis is effectively initiated, underscoring the efficiency and effectiveness of this treatment approach. Although a lower cumulative hCG dose was observed in the group A, reverse causality cannot be excluded. This finding may be interpreted as exploratory. The median time to spermatogenesis was 12 months, with one participant achieving it as early as 4.5 months. While the median duration for groups A and B was 12 months, it was slightly longer in group C at 15 months, but this difference was not statistically significant. Previous research has indicated a wide range in the time taken to initiate spermatogenesis, typically spanning from 3 months to 24 months, influenced by treatment protocol, duration and patient compliance (15, 25, 26).

The study objectively described the improvement in various aspects, which included virilization, sexual functioning and QoL.

BHS was utilized to objectively evaluate virilization, and it was monitored every 3 months in all participants. Notably, no prior study had reported an objective scoring method for assessing virilization in HH patients. At the start of the trial, scores were comparable in all the groups. However, significant intergroup comparisons of interval changes at various time points revealed the most substantial improvement in group A. The incorporation of exogenous T into the conventional treatment regimen yielded favorable outcomes in terms of virilization progression, evident as early as 3 months into treatment. This improvement was directly associated with maintaining plasma T levels within the normal range among participants in Group A. In contrast, participants in the other two groups experienced a delayed response, with T levels beginning to rise only after at least 3 months of treatment. By the ninth month, levels had normalized; however, this delay posed challenges. Similarly, Lee et al. conducted a study on CHH patients and noted that serum T started to rise after 3 months of therapy (21).

The prolonged timeframe for normalization in groups B and C contributed to reduced treatment adherence. Participants often reported dissatisfaction due to the absence of noticeable physical improvements in the early stages of therapy. This lack of visible progress not only diminished their motivation to continue the regimen but also had adverse psychosocial effects, impacting self-esteem and overall treatment satisfaction. Therefore, timely normalization of T levels appears to play a critical role in sustaining adherence and achieving favorable psychological and physical outcomes. Although plasma T levels eventually became comparable across all groups, early virilization had a marked positive impact on quality of life. This finding holds important clinical relevance, as early improvements contributed significantly to patient compliance, a crucial factor in a treatment regimen characterized by multiple injections and high costs.

Sexual dysfunction has an impact on both the psychosocial and emotional state of any person, and delayed normalization leads to poor compliance (20). Therefore, the scoring system for sexual functioning assessments like SDI-2 was explored. These have been used in assessing both congenital and acquired causes of hypogonadism (27, 28). The assessment among the groups at different time intervals significantly improved in group A, and by the end of one year, the increase was 178.3% in group A. This was the first time where this scoring system had been applied for monitoring CHH on treatment.

The improvement in QoL was assessed with self-reported rating scales namely PDS and qADAM questionnaires. Intergroup comparison of PDS showed a significant improvement at 3 months in group A (p <0.001), and as expected increase trend was highest in group A at all the time points. Similarly, the qADAM questionnaire also had significant improvement in all the time points (p value at 3 and 6 months <0.001, 12 months 0.006). PDS, an easy self-reported measure of physical development had shown to have a good correlation with the gold standard pubertal rating i.e. Tanner and Marshall staging (29). The qADAM questionnaire was able to quantify the severity of testosterone deficiency in the elderly.(23) These were used as an indirect measure of symptom improvement with therapy. Few studies on QoL assessment on CHH had been described using complicated multiple steps requiring measures and observed to have poor health-related QoL (30, 31). In contrast, our assessment was a comparatively easy way of assessing QoL, and as anticipated at baseline all had poor scores which increased significantly with time.

A meta-analysis had shown that prior T therapy did not affect the spermatogenesis in CHH patients. However, it is important to note that none of the studies included in the meta-analysis were randomized control trials (32). This aspect was addressed in the study, which demonstrated that it did not affect the induction of spermatogenesis. Despite older age in the participants with the prior testosterone group, spermatogenesis did not appear to be impaired.

Additionally, the study also explored the utility of new monitoring markers like AMH and Inh B. Baseline AMH levels were similarly high across the groups (p=0.945). With treatment, the expected fall in AMH corresponded to the Tanner stage, and all groups achieved the target. AMH is produced by immature Sertoli cells and is downregulated during pubertal maturation under the influence of rising ITT acting via androgen receptors. AMH serves as a sensitive marker of both gonadotropin and androgen action within the testis, reflecting the functional state of Sertoli cells. The suppression of AMH therefore signifies androgen-mediated Sertoli cell maturation, a prerequisite for the establishment of spermatogenesis. Although AMH is not a direct measure of spermatogenesis, its decline provides a reliable surrogate for spermatogenesis, particularly in contexts where direct assessment of intratesticular androgen activity is not feasible (33). Thus, targeting AMH <7.4 ng/ml was suggested, as the spermatogenesis rate in group A was not compromised. Further analysis of AMH as a predictor of spermatogenesis did not yield significant results, indicating that spermatogenesis depends on multifactorial factors. Inh B has been extensively investigated across multiple studies as the marker of spermatogenesis, owing to its association with functional Sertoli and germ cell numbers (15, 34). In the study, the median Inh B level was measured at 129 pg/ml among participants who achieved spermatogenesis. Notably, another study has indicated that an Inh B level of 60 pg/ml predicts spermatogenesis. Similarly, the study revealed that a follow-up cut-off of 66.8 pg/ml exhibited a sensitivity of 92.6% and a specificity of 100% for predicting spermatogenesis (15). Therefore, Inh B could be considered a well-established marker as a predictor of spermatogenesis, but AMH needs further exploration.

Other factors associated with spermatogenesis were also explored, and USG mTV (p=0.026) and hCG dose (p=0.004) at the time of spermatogenesis were observed to have significant results. The USG mTV cut-off of 1.97 ml demonstrated a sensitivity of 86.2% and a specificity of 62.5%. This value corresponded to TV by an orchidometer of just over 4ml (33). It is crucial to consider this information, as other studies have indicated that a TV of more than 8 ml predicts spermatogenesis (35). In our cohort, earlier normalization of testosterone levels allowed semen samples to be obtained sooner, enabling earlier documentation of spermatogenesis. In prior studies, delayed testosterone normalization led to later evaluation of spermatogenic outcomes. The hCG dose of 9000 IU/week showed a sensitivity of 79.3% and a specificity of 87.5% for indicating spermatogenesis. This dose was higher than the median dose in group A participants, therefore, concomitant T along with hCG and FSH can decrease the dose even further.

The most common side effect observed was gynecomastia (20%) with other side effects included acne and allergy to the drugs. Gynecomastia was more common in group B and C participants which could be explained by higher doses of hCG leading to enhanced aromatase activity. None of the participants experienced erythrocytosis and transaminitis requiring modification of the therapy. Gynecomastia was the most common side effect observed in other studies. But, none of these side effects led to the discontinuation of the treatment.

### Strengths and limitations of the study

The strength of the study was the design of the study being a randomized control trial. The use of various scoring systems to quantify the improvement in physical, sexual and QoL made this study a robust one. This study has important limitations. The modest sample size limits statistical power. Due to concerns regarding model stability, comprehensive multivariable adjustment was not performed, and residual confounding cannot be excluded. Larger, adequately powered studies are warranted. AMH as the treatment monitoring modality need further exploration in future studies. Another limitation was the non-blinded nature of the study, which could be a possible source of bias.

## Conclusion

With triple therapy, spermatogenesis is achieved in 84.6% of participants with a median hCG dose of 7500 IU/week. Objective monitoring of puberty, including virilization, sexual function, and quality of life, revealed broad improvements. Notably, quality of life significantly benefited without compromising spermatogenesis, suggesting triple therapy as a comprehensive approach to managing CHH.

## Data Availability

The raw data supporting the conclusions of this article will be made available by the authors, without undue reservation.
