# Supplementary material for: Comparing the response of triple therapy and conventional treatment in male congenital hypogonadotropic hypogonadism: a randomized controlled trial
Source: Front Endocrinol (Lausanne). 2026 Apr 1;17:1777584. doi: 10.3389/fendo.2026.1777584 (PMC13078972; doi:10.3389/fendo.2026.1777584)
Supplement: Supplementary file 1 [file DataSheet1.docx]

Supplementary Table 1: primary outcome

| **GROUP** | **A** | **B** | **C** | **p value** |
| --- | --- | --- | --- | --- |
| **hCG (IU/week)** | 7500  (6000-9000) | 9000  (8250-12000) | 9000  (7500-10500) | **0.016** |
| **FSH (IU/week)** | 450  (450-450) | 450  (450-450) | 450  (450-450) | 0.368 |
| **Time for spermatogenesis**  **(months)** | 12(9-15) | 12(10.5-15) | 15(12-16.5) | 0.345 |

Supplementary Table 2: Secondary outcome

| Group | A | B | C | p value |
| --- | --- | --- | --- | --- |
| BHS | 29(27-31.5) | 18(14.5-22) | 19(12-25.5) | 0.001 |
| PDS | 17(17-18) | 13(11-15) | 13(11-15.5) | <0.001 |
| qADAM | 40(37-40.5) | 35(32-38) | 32(30-34) | 0.002 |
| SDI-2 | 75(59.5-78) | 41(35-52.5) | 42(39-45.5) | <0.001 |
| HGS(kg) | 39(34.8-39) | 28(26.2-35) | 27.5(21.8-32) | 0.003 |
| BMD L1-4(g/cm^2^) | 0.858±0.964 | 0.855±0.124 | 0.809±0.109 | 0.522 |
| BMD FN(g/cm^2^) | 0.749±0.148 | 0.818±0.093 | 0.750±0.119 | 0.520 |
| BMD Rad 1/3^rd^(g/cm^2^) | 0.679±0.062 | 0.679±0.684 | 0.612±0.047 | 0.034 |

Supplementary Table 3: Comparison of participants with prior testosterone (T) therapy

| Prior T therapy | Yes (n=16) | No(n=29) | p value |
| --- | --- | --- | --- |
| Age | 29.5±5.98 | 24 ±5.98 | **0.006** |
| BMI | 21.9±4.79 | 22.6± 4.63 | 0.291 |
| LH | 0.3590.2-0.51) | 03(0.3-044) | 0.969 |
| FSH(baseline) | 0.57(0.35-0.820 | 0.54(0.3-1.12) | 0.784 |
| T | 0.31(0.09-0.83) | 0.49(0.09-0.66) | 0.413 |
| AMH | 18.7(10.1-25.3) | 18.4(11.7-24.8) | 0.692 |
| Inh B | 18.4(12.3-31) | 15.2(10.6-22.2) | 0.48 |
| E2 | 5(5-5) | 5(5-5) | 0.429 |
| mTV | 2(2-2.88) | 2(1-3) | 0.862 |
| USG mTV | 0.66(0.38-0.86) | 0.63(0.32-1.06) | 0.934 |
| hCG dose | 4000(4000-4000) | 4000(4000-4000) | 0.906 |
| FSH dose | 225(225-225) | 225(225-225) | 1 |
| Spermatogenesis (%) | 13(81.3) | 16(55.3) | 0.273 |
| At spermatogenesis |  |  |  |
| FSH | 3.63(2.45-4.39) | 2.98(2.12-3.45) | 0.155 |
| T | 18.8(7.14-23.8) | 18.1(10.4-25.6) | 0.975 |
| AMH | 2.9(1.8-4.26) | 3.78(2.25-6.16) | 0.133 |
| Inh B | 93(67.9-228) | 96.8(45.1-159.5) | 0.759 |
| E2 | 50.7(24-56.4) | 25.4(11.5-51.5) | 0.216 |
| mTV | 8(6-9) | 8(5.75-9) | 0.68 |
| USG mTV | 2.75(1.97-3.81) | 3.03(1.77-5.07) | 1 |
| hCG dose | 9000(7500-12000) | 9000(7500-12750) | 0.843 |
| FSH dose | 450(450-450) | 450(450-450) | 0.178 |

Supplementary Figure S1: Time profile graphs of a)testosterone, b)FSH, c) AMH and d)Inhibin B


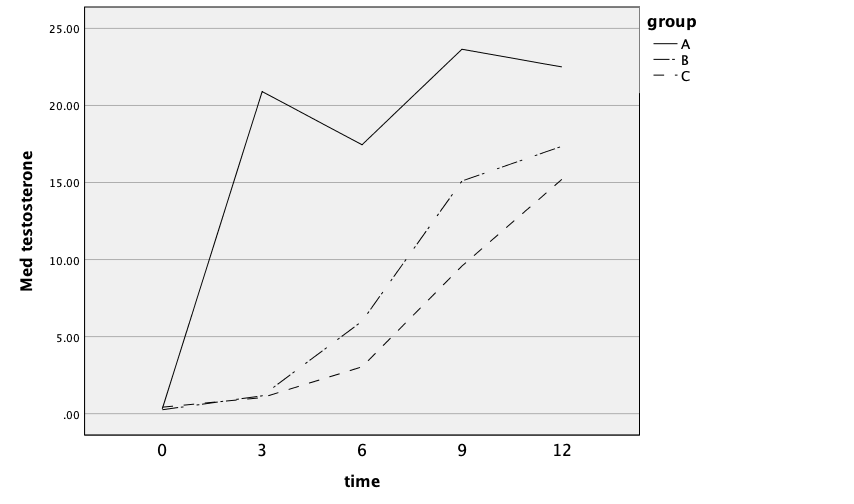


Figure S1(a)


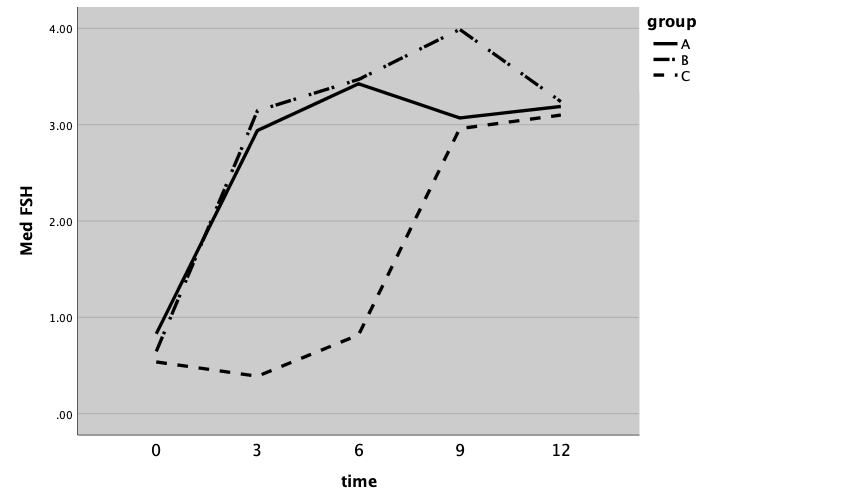


Figure S1 (b)


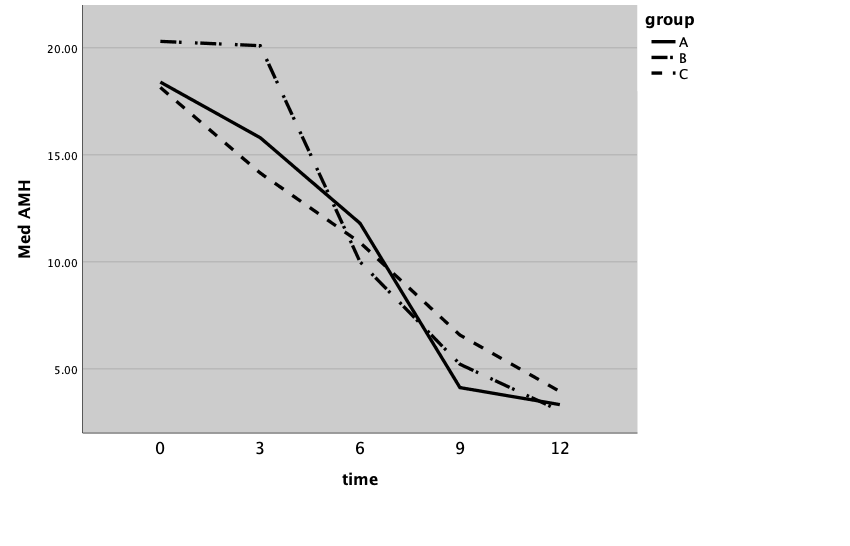


Figure S1 (c)


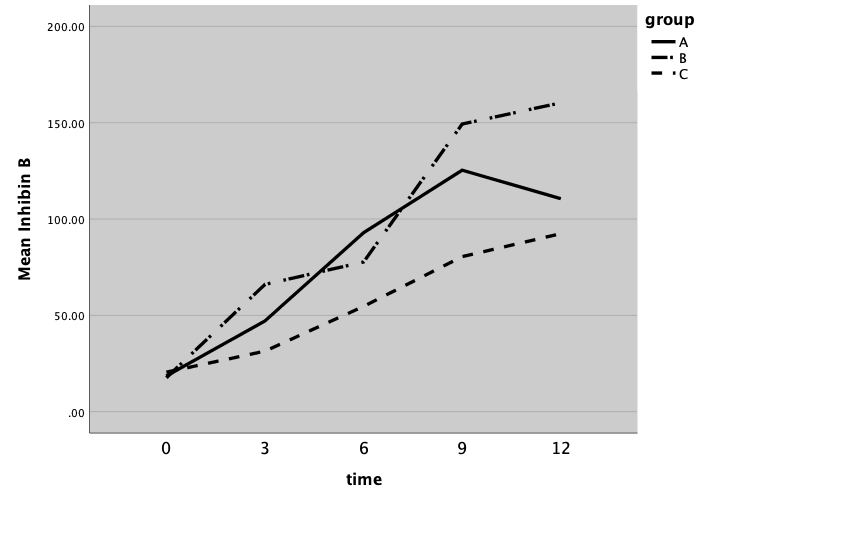


Figure S1 (d)

Supplementary Figure S2: Receiver operating characteristics (ROC) curve (2a) mean ultrasound testicular volume(USG mTV), (2b) hCG dose and (2c) Inhibin B(Inh B)


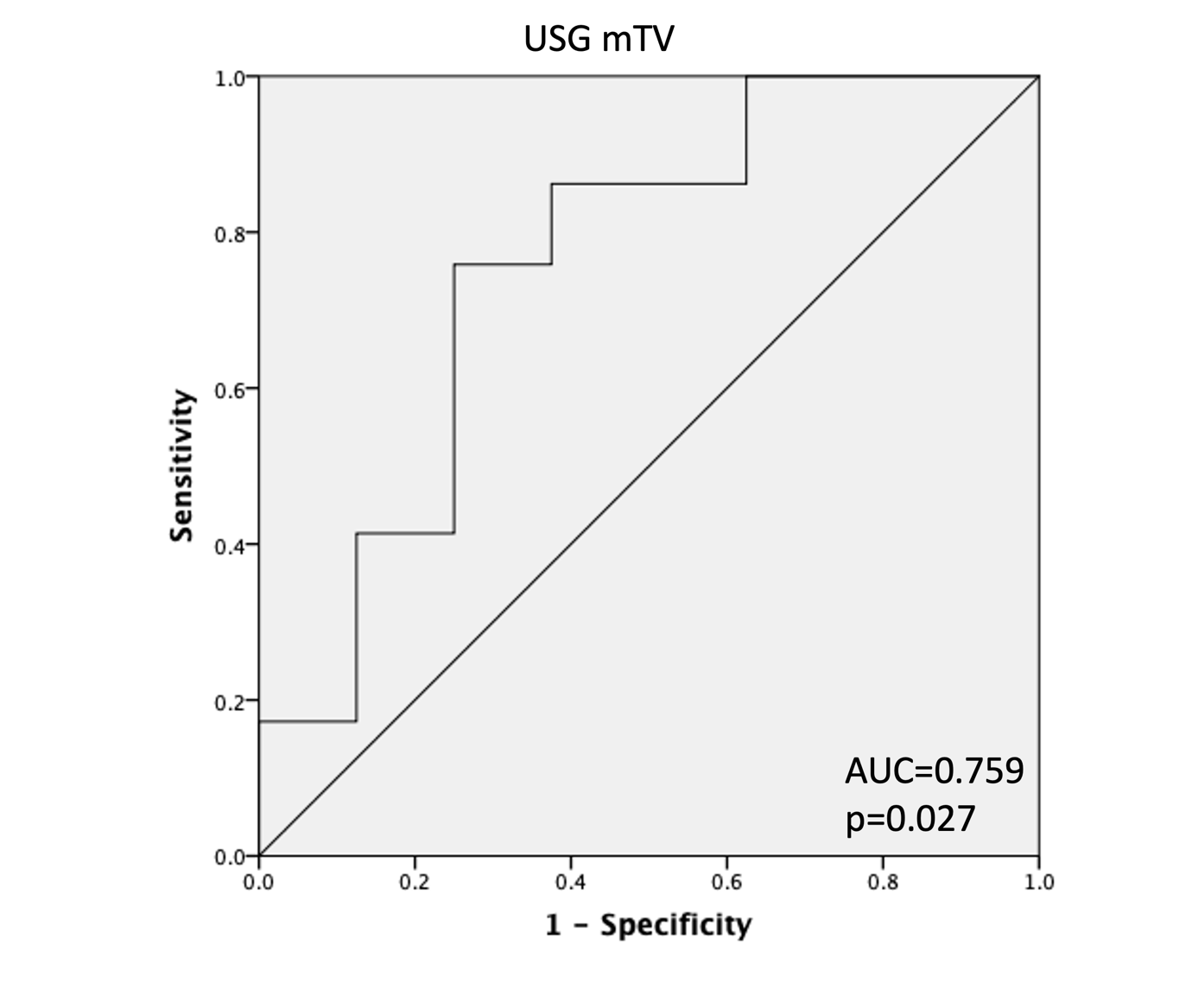


Figure S2a


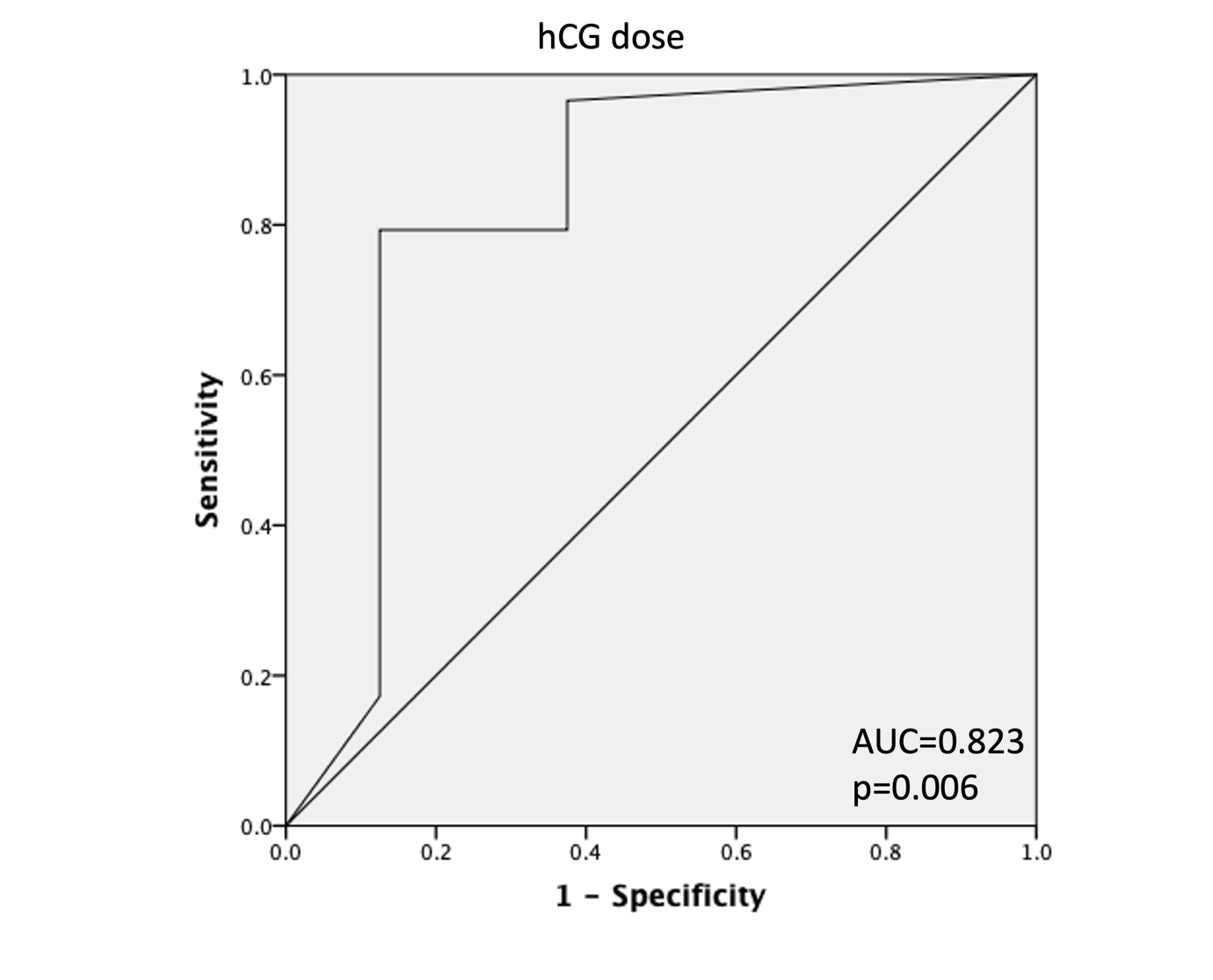


Figure S2b


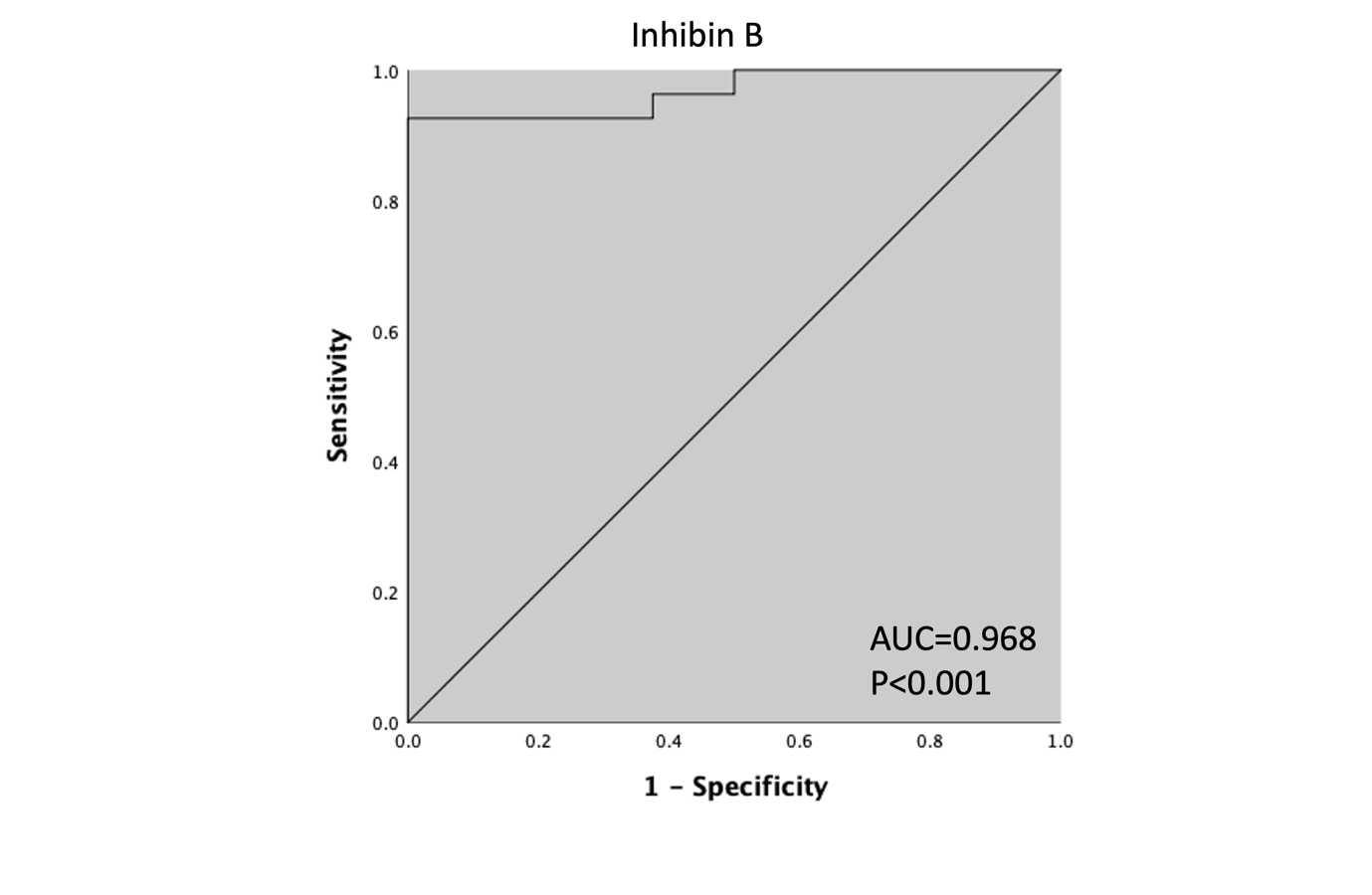


Figure S2c

BHS


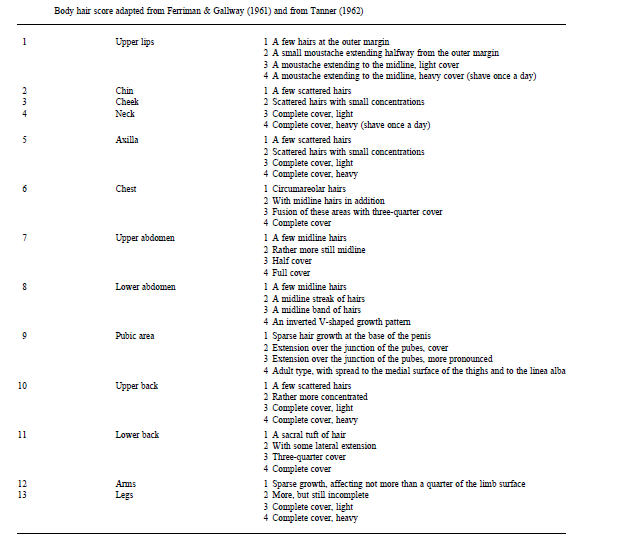


PDS

qADAM


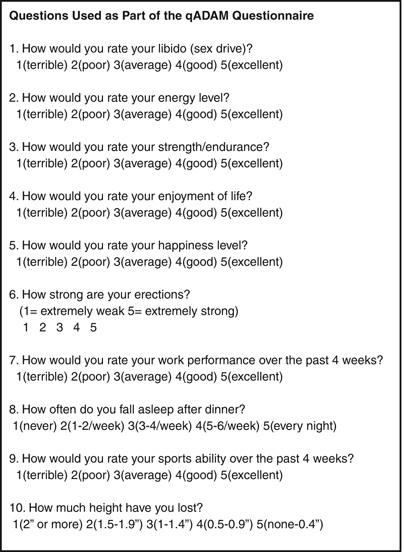


SDI-2

References values of hormonal profile

| S No. | Serum Hormone | Reference Values |
| --- | --- | --- |
| 1 | Testosterone | 9.9 – 27.8 nmol/l |
| 2 | FSH | 1.5-12.4mIU/ml |
| 3 | LH | 1.7-8.6mIU/ml |
| 4 | Estradiol | 7.63-42.6pg/ml |
| 8 | Prolactin | 4.0-15.2ng/ml |

Serum AMH reference range

| AGE | RANGE (ng/ml) |
| --- | --- |
| <14 days | 35-140 |
| 15 days-6 months | 55-210 |
| 6 months-2years | 85-320 |
| 2-9 years | 55-250 |
| 9-18 years |  |
| Tanner 1 | 35-200 |
| Tanner 2 | 10-140 |
| Tanner 3 | 4-55 |
| Tanner 4 | 4-22 |
| Tanner 5 | 4-21 |
| Adults | 3-18 |
